# Nanoparticles Targeting Innate Immune Cells in Tumor Microenvironment

**DOI:** 10.3390/ijms221810009

**Published:** 2021-09-16

**Authors:** Hochung Jang, Eun Hye Kim, Sung-Gil Chi, Sun Hwa Kim, Yoosoo Yang

**Affiliations:** 1 Center for Theragnosis, Biomedical Research Institute, Korea Institute of Science and Technology (KIST), Seoul 02792, Korea; hjang@kist.re.kr (H.J.); ehkelly@kist.re.kr (E.H.K.); 2 Division of Bio-Medical Science and Technology, KIST School, Korea University of Science and Technology, Seoul 02792, Korea; 3 Department of Life Sciences, Korea University, Seoul 02841, Korea; chi6302@korea.ac.kr

**Keywords:** nanoparticles, innate immune cells, tumor microenvironment, cancer therapy

## Abstract

A variety of innate immune cells such as macrophages, dendritic cells, myeloid-derived suppressor cells, natural killer cells, and neutrophils in the tumor microenvironments, contribute to tumor progression. However, while several recent reports have studied the use of immune checkpoint-based cancer immunotherapy, little work has focused on modulating the innate immune cells. This review focuses on the recent studies and challenges of using nanoparticles to target innate immune cells. In particular, we also examine the immunosuppressive properties of certain innate immune cells that limit clinical benefits. Understanding the cross-talk between tumors and innate immune cells could contribute to the development of strategies for manipulating the nanoparticles targeting tumor microenvironments.

## 1. Introduction

Based on the important roles of the various components of tumor microenvironment (TME) involved in tumor progression, strategies to therapeutically modulate the TME have recently emerged as a promising approach for the cancer therapy [1,2,3,4]. TME consists of stromal cells such as fibroblasts, extracellular matrix (ECM), immune cells including T and B lymphocytes, tumor-associated macrophages (TAMs), and natural killer cells [5]. The representative characteristics of TME are as follows: (1) vasculature abnormality due to an imbalance between pro-angiogenic and anti-angiogenic factors in tumor sites; (2) hypoxia caused by aberrantly aggressive cancer cell growth; (3) an acidic pH condition by glycolysis of cancer cells; and (4) residence of immunosuppressive cell types (TAMs, myeloid-derived suppressor cells (MDSCs), and regulatory T cells (Tregs)) [6].

In particular, the recent accumulating studies have focused on the relationship between innate immune cells and tumors. The innate immune system originally serves as an initial border of defense against foreign invaders and alerts the adaptive immune system of impending attacks [7]. In TME, a large number of innate immune cells indirectly influences tumor progression by controlling T-cell functions [8,9]. For example, type-2 (M2) TAMs express multiple immunosuppressive and tumor promoting factors, such as prostaglandin E2, vesicular endothelial growth factor, and IL-10, leading to suppressed anti-tumor responses [10]. In addition, the maturation of dendritic cells (DCs) elicits T-cell-mediated anti-tumor immunity. DCs specialized in local uptake of antigen from dying cancer cells, migrate to the draining lymph nodes and present the antigens to naïve T cells, supporting their differentiation into cytotoxic T cells [11]. In addition to participate in recruiting and activating adaptive immunity, the innate immune system is also involved in tumor suppressive activity, directly affecting tumor growth [12]. For example, natural killer cells (NKs) can recognize tumor-derived antigens or cell surface molecules, and lyse tumor cells [13]. Moreover, macrophages and polymorphonuclear granulocyte-like neutrophils are able to mediate anti-tumor responses via the antibody-dependent cellular cytotoxicity (ADCC) or antibody-dependent cellular phagocytosis (ADCP) [12]. Therefore, addressing the innate immunity will provide an attractive therapeutic opportunity to improve the efficacy of cancer treatments.

Many works were made to target adaptive immunity, such as the use of antibodies against immune checkpoints, such as PD-1, PD-L1 and CTLA4 (immune checkpoint blockades, ICBs), or chimeric antigen receptor-T (CAR-T) cell therapy [14,15,16]. Despite the numerous advantages of targeting innate immune cells in TME, however, the selective regulation of innate immune cells is still in its infancy.

The specific and efficient delivery of modulators to innate immune cells can improve the efficacy of cancer therapy. Nanoparticles (NPs), a type of material with a diameter of approximately 10 to 400 nm, can be decorated with functional moieties (e.g., targeting ligands for cancer cells) or be used to encapsulate therapeutic agents [17]. The representative advantages of cancer therapeutic strategies using NPs are as follows: (1) small sized enough to be diffused/absorbed into the body; (2) multiple delivery of targeting and therapeutic agents; (3) overcoming biological barriers; and (4) sustained or stimulus-triggered drug release [18,19]. Thus, many research groups are designing the NPs to increase the efficacy on anti-cancer therapy by targeting the components of TME and converting the immunosuppressive activity of TME [6]. Various nanomaterials, such as lipid-based NPs polymeric nanoparticles, inorganic NPs, and extracellular vesicles were designed as carriers for small molecules, nucleic acids, and proteins [20]. Although detailed in the next sections, each type of NP has its own characteristics. Briefly, (1) lipid-based NPs have simplicity for formulation and high bioavailability; (2) polymeric NPs show payload flexibility and easy surface modification; (3) inorganic NPs possess unique physical (electrical, magnetic, and optical) properties and tunable size; and (4) extracellular vesicles have the same membrane topology with the origin cell and low immunogenicity (Table 1) [21,22]. Thus, combining the aforementioned advantageous of NPs as a delivery molecule with type-dependent properties of NPs can overcome the limitation of traditional immune cell-modulating therapeutic strategies.

The general advantages of using NPs to target immune cells are relatively consistent with those of NPs as delivery molecules. In case of targeting DCs with NPs, NPs can be employed for delivering antigen-associated molecules (e.g., model antigen peptides) and adjuvants. This is able to promote long-term immune response against cancers via DC-based adaptive immunity [23]. In TME, it was well-known that TAMs play a pivotal role in various tumor cell biology, such as tumor cell proliferation, angiogenesis, metastasis, and immunosuppression [24,25]. Therefore, TAM targeting using NPs is also being studied a lot. Depending on the therapeutic agents loaded in and the functional moieties attached on NPs, there are 4-major TAM-modulated anti-cancer effects mentioned in the section below. These strategies facilitate comprehensive anti-cancer effects by not only depleting TAM, but also reprogramming pro-tumoric TAM. Accumulating evidences provide a rationale to utilize nanomaterials for the modulation of innate immune cells in TME. In particular, nanomaterials enable us to challenge the low selectivity, poor solubility, and high toxicity of certain chemotherapeutics targeting various immune cells [26,27]. In this paper, we review the recent researches that have used various NPs to control innate immune cells in TME, including TAMs, DCs, MDSCs, NK cells, and neutrophils.

## 2. Nanoparticles for Targeting TAMs

Macrophages are major innate immune cells which play critical roles as an immune regulator. In particular, macrophages can participate in anti-tumor immune responses via phagocytosis, the production of inflammatory cytokines, and the attraction of other immune cells. Therefore, macrophages could be a key promising target for a cancer immunotherapy.

With featuring their phenotypes and remarkable plasticity, macrophages display two different subtypes that are a double-edged sword: M1-like phenotype and M2-like phenotype macrophages in the TME [28,29]. According to the classification, macrophages usually have their own properties that can contribute to inhibiting or activating tumorigenesis [30]. Pro-inflammatory M1-like macrophages, differentiated by lipopolysaccharide (LPS) and interferon-γ (IFN-γ), exhibit the ability of anti-tumor functions to secrete the pro-inflammatory cytokines (e.g., TNF-α, IL-6, IL-12, and IL-12) [31,32]. In contrast, pro-tumorigenic M2-like macrophages activated by IL-4 and IL-13 promote tumor progression and release anti-inflammatory cytokines (e.g., TGF-β, Arginase-I and IL-10) [33]. TAMs refer to macrophages in tumors, most of which express pro-tumorigenic M2-like macrophages. Hence, TAMs, which are primarily occupied by M2-like macrophages, are an attractive target for suppressing tumorigenesis in cancer immunotherapy.

**Figure 1:**
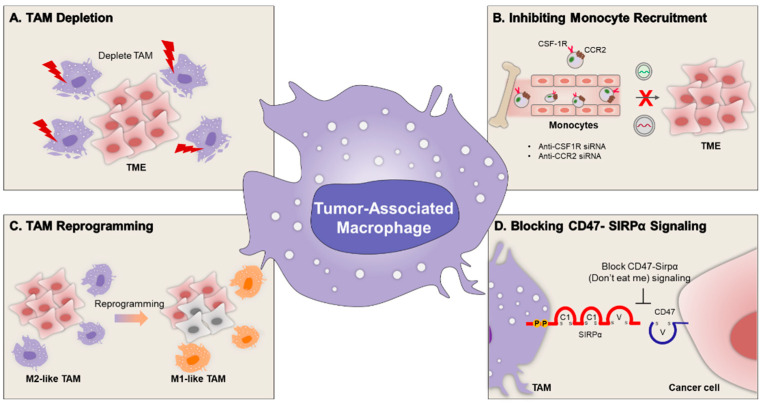
Nanoparticle-based strategies targeting tumor-associated macrophage in the tumor microenvironment. (**A**) Depletion of macrophages in tumor tissues; (**B**) inhibition of monocyte recruitment; (**C**) reprogramming of M2-type TAM to anti-tumoric M1-type macrophage; and (**D**) blockade of CD47-Sirpα signaling.

Here we introduce several NP-based strategies that target TAMs for suppressing tumor progression: (1) TAMs depletion; (2) inhibiting monocyte recruitment; (3) TAM reprogramming; and (4) blocking CD47- SIRPα signaling (Figure 1, Table 2) [34,35].

### 2.1. TAMs Depletion

Depletion of TAM eliminating pro-tumorigenic M2-like macrophages is a very potential strategy to treat a variety of cancer types. There are several studies that have effectively developed NPs as cancer treatments. Synthetic NPs (inorganic NPs or polymer-based NPs) are also considered as the most promising types for TAM depletion due to their characteristics that are easy to be engineered. Recent studies showed that the utilization of dendrimer NPs carrying the chemotherapeutic methotrexate that specifically recognize the folate receptor-2 (FOLR2) increases therapeutic efficacy by depleting TAMs. Since TAMs overexpress FOLR2, it can target macrophages to inhibit angiogenesis and improve anti-tumor effects. A dendrimer nanoparticle combined with the chemotherapeutic methotrexate (G5-MTX Nps) could improve TAMs targeting and alleviate the cancer development [36]. Tian et al., also built a nanoplatform using calcium bisphosphonate (CaBP-PEG) NPs to deplete TAMs in TME, which is synergistic for cancer radioisotope therapy [37]. Interestingly, the use of CaBp-PEG NPs is a powerful strategy for drug delivery due to its degradable nature in weak acidic TME. Therefore, CaBp-PEG NPs are attractive biocompatible and biodegradable nanoplatforms for the delivery of therapeutic drugs. Gold NPs are also applied to this relevant study. For example, Kim et al. suggested that gold NPs combined with the CD163 antibody on the silica surface targeting the depletion of M2-like TAMs could be used as an effective approach to increase the proportion of M1-like TAMs and enhance anti-tumor effects [38].

### 2.2. Inhibiting Monocyte Recruitment

The second strategy of TAM targeting is to inhibit the recruitment of macrophages derived from blood monocytes. This approach is to block specific signaling and tumor-derived chemotactic signals [39]. The colony-stimulating factor-1 (CSF-1), also known as macrophage colony-stimulating factor (MCSF), contributes to the survival and differentiation of macrophages and also regulates monocyte recruitment. Owing to its key role as a regulator in the survival of monocyte, CSF-1 and its receptor CSF-1R were also studied as therapeutic targets [40]. Qian et al., developed the dual-targeted lipid NP-delivering anti-CSF-1R small interfering RNA (siRNA) to inhibit the CSF-1/CSF-1R signaling axis [41]. In particular, the lipid-based NPs show higher efficiency for penetration in a solid tumor using biocompatible fusion peptides. Moreover, anti-CSF-1R siRNA could affect the survival of M2-like TAMs by blocking their recruitment and thus reduce the tumor volume in melanoma models. The chemokine ligand 2 (CCL2) and its main receptor CCR2 signaling pathway are also key factors in targeting macrophage recruitment. In various cancer models, the signaling pathway of CCL2/CCR2 axis are upregulated, leading to tumorigenesis [42]. Thus, the strategy of targeting CCL2/CCR2 signaling is an attractive approach to mediating macrophage recruitment properties. The utilization of cationic polymeric NPs carrying CCR2 siRNA could block the expression level of CCR2 in monocytes, suppress monocyte recruitment in tumor tissues, and enhance anti-tumor effects in the breast cancer model [43]. Among the metal-based inorganic NPs, silver NPs (AgNPs) are being used for producing medical supplies based on their inherent anti-bacterial/fungal/viral properties [44,45,46]. With the growth of the field of inorganic NPs, several research groups are studying the effects of AgNPs on the immune system, and it is reported that AgNPs have an adjuvant effect inducing recruitment and activation of local macrophage [47].

### 2.3. TAM Reprogramming

TAM depletion and inhibition of macrophage recruitment have the disadvantage of losing powerful immune modulators called antigen presenting cells (APCs). Therefore, recently, reprogramming TAM from M2-like macrophages to M1-like macrophages has attracted attention as a strategy for cancer therapy. One study has shown that M0 and M2 macrophage were converted to M1 macrophage associated with anti-tumor effects using drug-free mannose decorative liposomes [48]. Mannosylated liposomes contributed to superior cellular internalization with optimal nanoparticle size and excellent biocompatibility. Due to M2 macrophage in TAMs with high levels of mannose receptors, mannose decorative liposomes that inhibit G422 glioma tumor growth could contribute to enhancing anti-tumor efficacy. The albumin-based delivery NP is another promising method to promote reprograming TAMs. Zhao et al., devised an albumin-derived nanoplatform that delivers both the disulfiram/copper complex and macrophage modulator regorafenib for reprogramming macrophage [49]. The designed albumin-based NPs enhance the solubility of hydrophobic drugs and provide the effective targeting strategy through the biomimetic delivery system. In terms of genetic reprogramming of macrophages, the use of poly-beta-amino-esters (PBAE) nano-vehicles with synthetic mRNA encoding interferon regulatory factor 5 (IRF5) was able to lower the proportion of M2-like TAMs and increase the percentage of M1-like TAMs [50]. The positively charged PBAE enhance synthetic mRNA stability with its biocompatibility and biodegradability.

Recently, extracellular vesicles which have a significant impact on tumor suppression have gained much interest. Particularly, extracellular vesicles, such as exosomes naturally derived from cells, exhibit targeting ability and, therefore, suitability as biomolecular carriers. For example, the use of exosome-mimetic nanocarriers derived from M1 macrophages could improve anti-tumor efficacy by reprogramming macrophages [51]. The other study also showed that using nano-sized vesicles derived from bone marrow-derived macrophages (BMDMs) results in reprogramming of TAMs from M2 to M1 pro-tumor macrophages to anti-tumor macrophages [52]. Additionally, NPs encapsulated with oligonucleotides, such as microRNAs (miRNAs) and siRNA, is another promising strategy for re-education of TAMs. For example, Xiao et al., developed a dual pH-sensitive NP delivering IKKβ siRNA and STAT6 inhibitor to repolarize of M2-like TAMs into M1-like TAMs [53]. In particular, pH-sensitive NPs with PEG shedding reprogramed M2-like TAMs to M1-like TAMs for cancer immunotherapy by targeting M2 macrophages using M2-targeting peptide.

### 2.4. Blocking CD47-Sirpα Signaling

Many recent studies have demonstrated that the inhibition of the CD47-signal regulatory protein-α (Sirpα) signaling is a promising target for activating macrophage phagocytosis. Representing a “don’t eat me” signal, CD47 is overexpressed on the surface of cancer cells in many tumor types [54]. When Sirpα from macrophages binds to CD47, it controls and blocks the activation of macrophage phagocytosis [55]. Therefore, the inhibition of the CD47-Sirpα signaling axis promotes macrophage phagocytic ability against tumor cells. For example, Rao et al., exhibited a cellular membrane coated with magnetic NPs that efficiently block CD47-Sirpα signaling and trigger macrophage-related anti-tumor immune responses [56]. Koh et al. also established an engineered exosome platform decorated with SIRPα variants on the surfaces [57]. Owing to decorating with Sirpα variants, engineered exosomes could target CD47-overexpressing cancer cells, regulate macrophage phagocytosis through disruption of CD47-Sirpα interaction and lead to innate and adaptive immune responses against cancer.

## 3. Nanoparticles for Targeting DCs

**Figure 2:**
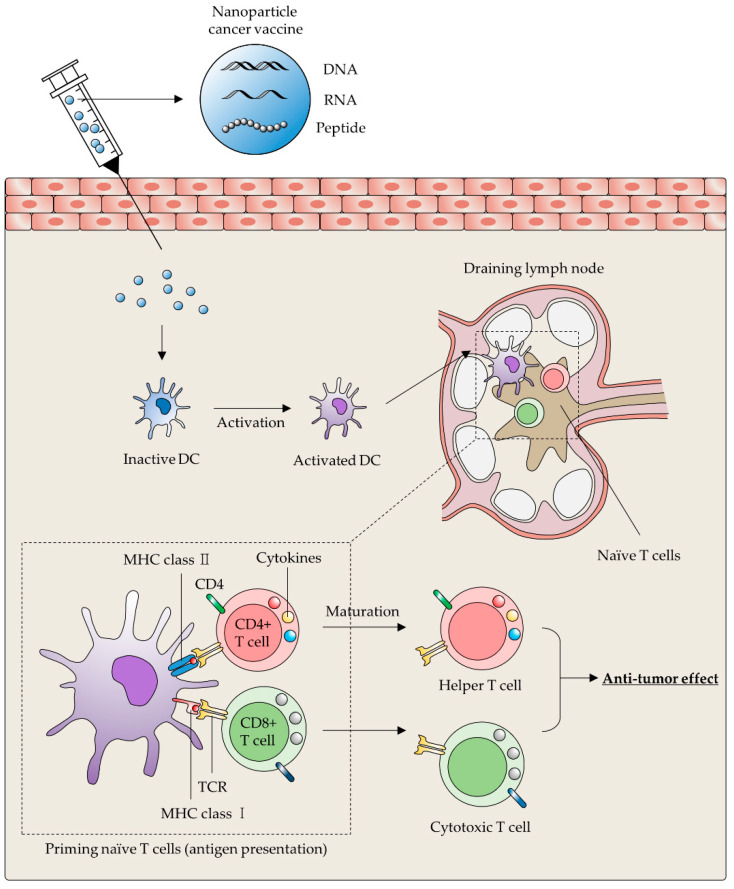
Schematic illustration presenting brief mechanism of DC-based cancer vaccine. After vaccination with TAA-related payloads encapsulating NPs, TAA can be delivered into DCs and processed. Naïve T cells (CD4+/CD8+) are primed through presented TAA peptides on MHC I/II molecules of activated DCs. Naïve CD4+/CD8+ T cells differentiated to the Th cell and Tc cell respectively. Functional Th and Tc cells attack specific tumor cells expressing TAA presented by DCs.

DCs, the most professional APCs in the immune system, are able to bridge the gap between innate and adaptive immune response [58,59]. Once DCs uptake specific antigens, they present the processed antigen peptide through the major histocompatibility complex I (MHC I) and the MHC II molecules to the CD8+T cells and CD4+ T cells in the lymphoid organs, respectively [58,59]. In addition to MHC-mediated antigen presentation, additional stimulation by costimulatory molecules, such as CD80, CD86 and CD40, are also required to decide orientate T-cell differentiation [60]. After completing the T-cell maturation process by DCs, CD4+ and CD8+ T cells are differentiated to the helper T cell (Th) and cytotoxic T cell (Tc) to establish the long-lasting therapeutic effect against the cancer cells (Figure 2) [23].

Given the aforementioned roles of DCs in the immune response, it facilitates assuming that DCs act as the “key-player” of cancer immunotherapy by capturing tumor-associated antigens (TAA) and interacting with T cells.

### 3.1. Cancer Vaccines

Considering the central role of DCs that induce priming and activating antigen-specific T cells in the immune system, DC-based cancer vaccines resulting in a potent anti-cancer immunity by DC maturation were studied over the past few decades [61,62]. Although the autologous DC-based cancer vaccine that has undergone ex vivo pulsing by specific TAA shows noteworthy therapeutic effects in some type of cancers, some drawbacks, such as (1) insufficient migration to the lymph nodes [63], (2) low blood concentrations [63], (3) labor intensity [64], and (4) high cost for vaccine preparation [65], still exist. Thus, many research groups have tried to develop the next generation of DC-based cancer vaccines that can induce maturation and activation of DCs in our body by delivering TAA and immunomodulators (adjuvants) [20,66].

In the field of drug delivery, NPs are considered as potent materials for delivering various molecules such as small molecules, nucleic acids, and peptides [67,68,69]. NPs have been typically employed as a carrier material for effective cancer vaccination because they protect premature TAA and adjuvants from enzymatic degradation in the body, and can be engineered with DC-targeting moieties [70,71]. In this section, we have summarized different types of NPs (Table 1 and Table 2) for inducing robust and long-lasting immune response against cancers.

**Table 1:** Different types of nanoparticles and their properties as a delivery molecule.

Categories	Types	Features	References
Lipid-based ^1^ NPs	Liposome	Amphiphilic structure Surface modification High bioavailability	[41,48,72,73,74,75]
	^2^ LNPs		
Inorganic NPs	^3^ AuNPs	Customizable size and structure Well-established application for clinical imaging	[37,38,47,56,76,77,78,79,80]
	Silica NPs		
	Selenium NPs		
	^4^ AgNPs		
	Calcium bisphosphonate		
	Iron NPs		
Polymer-based NPs	Polymeric micelle	Biocompatibility Biodegradable Surface modification Payload flexibility (hydrophilic/hydrophobic)	[36,43,50,81,82,83,84]
	Dendrimer		
	Cationic polymeric NPs		
	Poly-β-amino ester		
Extracellular vesicle	Extracellular vesicle	High biocompatibility Low immunogenicity Low immune clearance Surface modification Payload flexibility Expression of native membrane proteins	[51,52,57,85,86]

^1^ NPs: nanoparticles; ^2^ LNPs: lipid nanoparticles; ^3^ AuNPs: gold nanoparticle; ^4^ AgNPs: silver nanoparticles.

**Table 2:** Overview of nanoparticles with payload and targeting moieties for cancer therapy.

Nanoparticle	Ligand/Target	Payload	Purpose	Reference
Lipid-based NPs	Mannose/Mannose receptor	-	TAM reprogramming	[48]
	α-^1^ M2pep/^2^ SR-B1	Anti-CSF-1R siRNA	Inhibiting monocyte recruitment	[41]
	-	^3^ OVA, Poly(I:C), ^4^ gp100, ^5^ TRP2	DC-based cancer vaccine	[72,74]
	T1 DNA aptamer	^6^ Dox	MDSC depletion	[75]
Inorganic NPs	^8^ FORL2	^9^ MTX	TAM depletion	[36]
	CD163 antibody/CD163	-		[38]
	-	CCR2 siRNA	Inhibiting monocyte recruitment	[43]
	-	RFP, CpG-ODN, OVA	DC-based cancer vaccine	[76]
	Sirpα/CD47	-	Blockade CD47-Sirpα signaling	[56]
	-	Dox, ^10^ ATRA, IL-2	MDSC depletion	[87]
	α-EGFR, α-4-1BB, and α-CD16	^7^ EPI	NK cell activation	[84]
Polymer-based NPs	Mannose/Mannose receptor	OVA, CCR7 pDNA	DC-based cancer vaccine	[82]
	-	OVA, CpG		[83]
	-	^11^ IRF5 mRNA	TAM reprogramming	[50]
Extracellular vesicle	-	STAT6 inhibitor, IKKβ siRNA	TAM reprogramming	[53]
	Sirpα variants/CD47	-	Blockade CD47-Sirpα signaling	[57]
	CD40L/CD40	-	DC-based cancer vaccine	[85]
	^12^ NKG2D ligand and IL-15Rα	-	NK cell activation	[86]

^1^ M2pep: M2 macrophage binding peptide; ^2^ SR-B1: a scavenger receptor B type 1; ^3^ OVA: ovalbumin; ^4^ gp100: glycoprotein 100; ^5^ TRP2: tyrosinase-related protein 2; ^6^ Dox: doxorubicin; ^7^ EPI: epirubicin; ^8^ FORL2: folate receptor-2; ^9^ MTX: methotrexate; ^10^ ATRA: all-trans retinoic acid; ^11^ IRF5: interferon regulatory factor 5; ^12^ NKG2D: natural killer group 2 member D.

#### 3.1.1. Lipid-Based Nanoparticle

Liposomes are self-assembling lipid-based NPs composed of amphiphilic phospholipids. Because liposomes have a hollow sphere structure that encompass hydrophilic central space with hydrophobic lipid bilayer, they can deliver both hydrophilic/lipophilic payloads simultaneously [88,89,90,91]. Since liposomes have a comparable structure to cell membrane in our body, they possess great biocompatibility and low cytotoxicity, which is advantageous in clinical application [88,89,90,91]. In addition, surface modification using many of the functional residues, such as DC-targeting moieties, is able to improve anti-cancer immunity by facilitating accurate antigen/adjuvants delivery to DCs [73].

Varypataki and colleagues demonstrated that the synthetic model antigen (OVA24)-engineered and poly (inosinic-polycytidylic acid) (poly(I:C))-adjuvanted liposomes significantly induced DC maturation followed by OVA24-specific CD8+ T-cell responses [72]. According to this study, incubation of DCs with OVA24/poly(I:C)-liposomes induced enhanced CD80 expression on DC surface in vitro. After immunization by intradermal injection of OVA24/poly(I:C)-liposomes to naïve C57BL/6 mice, authors confirmed that significantly improved antigen-specific CD8+ T-cell activation [72].

Lipid nanoparticles (LNPs) are another subset of lipid-based NPs with structural differences from conventional liposomes. Whereas traditional liposomes are composed of one or more lipid bilayers encompassing a hydrophilic inner space, LNPs form multiple micelle-like structures within their particle core depending on formulating methods [92]. In general, LNPs are widely used for the delivering a variety of nucleic acids due to their major components, such as ionizable lipids. The ionizable lipids have a positive charge or a neutral form at low pH or physiological pH, respectively. These ionic features of lipids allow efficient complexation with negatively charged nucleic acids, facilitating not only intracellular delivery but also endosomal escape of the payloads [21,74,93,94,95,96].

According to the experimental results from Blankschtein’s group, it was found that a single immunization with LNPs containing OVA mRNA induced a strong cytotoxic CD8 T-cell activation. Moreover, significant tumor growth inhibition was also confirmed after treatment of LNPs possessing mRNA encoding glycoprotein 100 (gp100) and tyrosinase-related protein 2 (TRP2) in the B16F10 melanoma implanted mice model [74].

#### 3.1.2. Inorganic Nanoparticles

As antigen/adjuvants carrier for DC-mediated anti-cancer immunity, inorganic NPs have some advantageous physicochemical properties such as customizing of size, shape, surface functionalization, and structural conformation [20]. Among these, gold NPs (AuNPs) are considered as one of the most suitable NPs. In addition to aforementioned features of inorganic NPs, AuNPs can also target the immune-associated organs, such as draining lymph nodes (LN) or the spleen, and be tracked by using computed tomography (CT) [76,97]. Because this nature of AuNPs facilitates confirming NPs reach the appropriate target, it gives us insight to the prognosis for the effect of cancer vaccination.

Indeed, Lee and colleagues reported that model antigen (red fluorescent protein (RFP)) and adjuvants (CpG ODN)-engineered AuNPs can convert naïve T cells to active ones, as well as promote antigen-specific T-cell proliferation after LN accumulation. In addition, Gulla et al. showed that AuNP-based nanoplex functionalized with a thiolate ligand (SGSH) and caused a long-lasting (for 180 days) immune response against the murine melanoma by delivering a melanoma antigen (MART1)-encoding DNA vaccine [76].

Another type of inorganic NP for improving the effect of DC-based cancer vaccination is the mesoporous silica nanoparticles (MSNs). Although MSNs possess negatively charged and hydrophilic silanol groups (Si-OH) that allow them to target LNs, MSNs showed low migration efficiency to LNs due to their relatively large size (>100 nm). To tackle this, the Sun group fabricated a smaller size of MSNs (around 80 nm) with different pore sizes, showing enhanced MSNs accumulation in LNs and anti-cancer effects [78].

A previous study from Song and colleagues suggested that polysaccharide-functionalized selenium NPs could be a novel therapeutic tool for lung cancer by modulating dysfunctional immune cells in malignant pleural effusion (MPE) [80]. The selenium, an essential trace element with anti-oxidant/toxic effects, is able to maintain cell membranes and protects lipids, lipoproteins, and DNA from oxidative damages [98,99,100]. In addition to these advantages, selenium was also known to possess favorable properties that drug delivery materials should have, such as low toxicity and the ability to penetrate biological barriers [80,101]. According to the experimental results in this study, SeNPs@LNT treatment boosted DC maturation from 14.5% to 30.8%. Indeed, the level of human leukocyte antigen (HLA)-A2 increased after incubation with SeNPs@LNT and an enhanced population of Tc cells was also observed.

Iron NPs are also considered as one of the most promising types of inorganic NPs for developing a DC-based cancer nanovaccine due to their biocompatible properties including biodegradability, circulation, and customizable structures [102]. In particular, the superparamagnetic iron oxide NPs (SPIONs) are being applied in various bio-medical fields as iron supplements [103], MRI contrast agents [104], and magnetic particle imaging (MPI) tracer [105]. Similarly to other types of NPs, it was well-known that SPIONs could be coated with various polymers and conjugated with anti-tumor molecules including tumor-specific antibodies and nucleic acids (siRNA/miRNA) [106]. For example, the Yuan group developed an OVA-conjugated SPION (Fe_3_O_4_-OVA) and confirmed its DC-stimulatory ability and anti-tumor effects via in vitro/vivo studies. After incubation with Fe_3_O_4_-OVA, a significantly enhanced secretion level of Th-1 biased cytokines from DC2.4 cells (TNF-α, IL-6, and IFN-γ) was detected compared to free Fe_3_O_4_ NPs and soluble OVA only groups. Moreover, it was confirmed that immunization with Fe_3_O_4_-OVA facilitated tumor growth inhibition in tumor-bearing mice [77].

According to a previous study published in 2018, Orlowski et. al., demonstrated that AgNPs have an immunological stimulating effect on DCs. In this study, the authors formulated tannic acid-modified AgNPs (TA-AgNPs) and treated it to JAWS II immature DCs cell line. After 24 h incubation with TA-AgNPs, JAWS II cells showed a significantly increased expression level of surface activation markers (MHCII, CD40, and CD86) and similar patterns are observed from follow-up experiments using BMDCs. Moreover, it was found that TA-AgNPs treatment induced antigen-specific T-cell activation in experiments with herpes simplex virus type 2 (HSV2) and TA-AgNPs [79]. Although there were no experimental results to evaluate anti-cancer efficacy of Ta-AgNPs using a cancer model, it is thought that Ta-AgNPs can be developed as a vaccine component targeting virus-related cancer (e.g., cervical cancer) because it was be able to induce DC-based adaptive immune responses.

#### 3.1.3. Polymer-Based Nanoparticles

Polymers not only play fundamental roles in producing conventional pharmaceutical formulations, but also act as building blocks for nanomaterial-mediated delivery systems due to their biocompatible and biodegradable features [20]. Depending on the synthesis methods, there are two representative types of polymeric NPs: (1) polymeric micelles with amphiphilic core/shell [107,108], and (2) dendrimer possessing hyper-branched structure [109,110].

The functional characteristics of micelles are based on amphiphilic polymer that gathers to form a central/shell (outer) structure in the solution state. The hydrophobic center serves to store hydrophobic molecules, and the hydrophilic outer encloses the hydrophobic center to impart water solubility to the NPs [111]. Thus, these unique structures and properties of polymeric micelles make it an effective carrier molecule for a drug delivery system. Yang and colleagues fabricated the mannose-modified synthetic micelle mainly composed of chitosan (CO), which are natural polysaccharide polymer, and steric acid (SA). Owing to the immune-stimulating activity of CO [112] and SA-based lipid structures that resemble cell membrane, synthetic COSA micelles were expected to be a carrier molecule with effective immune adjuvant effects [82]. According to this study, mannose-engineered COSA (M-COSA) micelles encapsulating OVA and CCR7-encoding pDNA (M-COSA/OVA/pDNA) showed significantly enhanced expression levels of CD40 and CD86 in DCs. In addition, M-COSA-based immune complexes induced direct migration of DCs to LN by CCR7 expression [82].

Dendrimers, another type of polymer NPs, were also studied as a carrier molecule for model antigen and adjuvants. According to the previous study for dendrimer-based biomolecule delivery, cationic dendrimer scaffold decorated with guanidinobenzoic acid (DGBA) facilitated protein/peptide binding, endocytosis and endosomal disruption resulting in efficient delivery of cargoes into the cytosol of living cells [113]. Follow-up study using DGBA from Xu et al. reported the great promises of DGBA-based NP for effective cancer vaccination [83]. Because of the high ability of DGBA in protein binding, the authors employed DGBA as a delivery vehicle for cancer vaccine-related components, such as OVA and cytosine-guanine dinucleotide (CpG), a TLR-9 agonist. OVA and CpG-engineered DGBA (DGBA-OVA-CpG) showed robust antigen-specific immune response and prophylactic effect against to B16-OVA melanoma [83].

#### 3.1.4. Extracellular Vesicles

Although many artificial NPs were widely used as a carrier molecule for therapeutic reagents, they have some biological hurdles such as inflammatory toxicity [114], immune recognition [115], and rapid clearance [116]. To overcome these obstacles, a number of studies using nano-sized extracellular vesicles (EVs) and exosomes, have gained attention. Because EVs are secreted from most cells in our body, they have a significantly great biocompatibility and relatively low immune clearance [22]. In addition, EVs can be engineered by transfecting the genetic vector-encoding specific functional moieties. EVs can express fully functioning naïve form of membrane proteins, not truncated or modified forms, due to the same membrane topology compared to their origin cells [117].

According to the previous study from Wang et al., exosomes secreted by CD40 ligand gene-modified tumor cell (CD40L-EXO) induced enhanced DCs maturation. After the bone marrow-derived DC (BMDCs) stimulation with CD40L-EXO, it was found that expression levels of MHC II, CD80, CD86, and CD40 were significantly increased. In addition, enzyme-linked immunosorbent assay (ELISA) assay for quantifying the cytokines in BMDC culture supernatant showed markedly increased levels of interleukin-12 (IL-12) that are associated to naïve T cell differentiation into helper T cells. After immunization using CD40L-EXO in mice, the increased production of anti-tumor-associated cytokines, including IL-2 and IFN-γ, were observed. Subsequently, enhanced tumor antigen-specific CTL response was also validated. Indeed, subcutaneously administered CD40L-EXO to tumor-bearing mice induced significantly reduced tumor size and an increased survival ratio of tumor mouse model [85].

The field of NP-based cancer vaccine has achieved the exceptional outcomes on anti-tumor effects. Given the aforementioned outstanding anti-tumor immune response of NP-based DCs cancer vaccines, developing optimized NPs for delivering TAA and adjuvants will give patients suffering from cancers new therapeutic options with specificity and safety.

### 3.2. DC Activation

Apart from employing the NPs as carrier molecules of vaccine-related components such as TAA and adjuvants, NPs per se can also be used to induce maturation of DCs. Representative characteristics of DC maturation are typically as follows: (1) production and secretion of inflammatory cytokines such as IL-6, IL-1β, TNF-α, and IL-12; (2) increased surface expression of both MHC and costimulatory molecules; and (3) chemokine responsiveness shift based on changes in chemokine receptor expression patterns [118,119,120].

According to the recent study from the Zhang group, fullerene derivatives, a type of carbon nanomaterial, are suggested as a potent stimulator for maturation of DCs. Similarly to the aforementioned NPs used for the cancer vaccine, gadolinium atom-entrapping fullerene derivatives (Gd@C82(OH)x) also have advantageous properties, such as biocompatible, water-soluble, and small size (approximately 25 nm average) [121,122]. After 48 h incubation with human DCs, [Gd@C82(OH)22]_n_ induced a significantly upregulated secretion level of pro-inflammatory cytokines. In addition, myeloid DCs incubated with [Gd@C82(OH)22]_n_ induced surface expression level of co-stimulatory (CD80, CD83, and CD86) and MHC (HLA-ABC and HLA-DR) molecules [121]. It is known that immature DCs normally express chemokine receptor type 5 (CCR5), whereas mature DCs have an increased expression level of chemokine receptor, CCR7 [119,123]. Indeed, stimulated DCs by [Gd@C82(OH)22]_n_ demonstrated converted chemokine responsiveness pattern from specific for CCL5 to CCL19 [121].

## 4. Nanoparticles for MDSCs Depletion

The accommodation of MDSCs at tumor tissues is correlated with suppression of T-cell proliferation or promoting the differentiation of regulatory T cells (Tregs). MDSCs are heterogeneous cell types of immature myeloid cells inducing immunosuppressive effects against cancers [124]. The representative immunosuppressive mechanisms of MDSCs can be divided into three categories. Briefly: (1) Depleting of ʟ-arginine by overexpressed arginase 1 and inducible nitric oxide synthase (iNOS) in MDSCs. Since ʟ-arginine is a requirement for T-cell proliferation and CD3 ζ-chain formation of TCR, the increased activity of arginase 1 and iNOS inhibits T cell proliferation and function [125,126,127,128]. (2) Induction of T-cell dysfunction by reactive oxygen species (ROS) and reactive nitrogen species (RNS) [129,130,131]. According to a previous study by Nagaraj et al., MDSCs-derived ROS molecule and peroxynitrite (the RNS produced from chemical reaction between superoxide radicals and nitric oxide (NO)) induce post-translational modification of TCR and CD8 molecules leading to antigen-specific tolerance of peripheral CD8+ T cells by disrupting binding affinity to phosphorylated MHC molecules [129]. (3) Interrupting anti-tumor immune response by promoting Tregs differentiation [124,132]. Tregs can inhibit immune function through inhibitory cytokines including IL-10, IL-35, and TGF-β [133,134]. Given the aforementioned immune-suppressive effects of MDSC, depleting or modulating MDSC should be a therapeutic strategy for cancer immunotherapy. In this section, we will handle the MDSCs-targeting NPs.

Liu et al. designed liposomes that functionalized with DNA thioaptamer (T1) for TME targeting. The results revealed that intravenously administered T1 aptamer accumulated in the tumor site, especially to PMN-MDSCs (CD11b+Ly6G+). In in vivo efficacy test using the orthotopic breast cancer mouse model, it was found that T1-functionalized and doxorubicin-encapsulating liposomes decreased PMN-MDSCs and induced infiltration of CD8+ T cells in tumor sites [75]. Thus, authors suggested that targeting and depleting intra-tumoral MDSCs could be a promising cancer therapeutic method.

In tumor-associated pathological conditions, various immune response-related molecules in TME such as IL-1β [135,136], IL-6 [137], prostaglandin E2 [138], VEGF [139], and IFN-γ [132] induce abnormal accumulation of immature myeloid cells by impairing the differentiation process. [140]. Thus, modulating the differentiation of MDSCs to appropriate immune cells can also be a considerable strategy for cancer therapy. Kong and colleagues introduced the modulating strategy for abnormal MDSCs differentiation using lipid-coated biodegradable hollow mesoporous silica nanoparticles (dHMLB). The authors co-delivered interleukin-2 as a T-cell growth factor and all-trans retinoic acid (ATRA), which can induce differentiation of MDSCs to mature DCs, macrophages, and granulocytes, using dHMLB (A/D/I-dHMLB) into the tumor-bearing mouse model. According to this study, the quantity of MSDCs in the tumor was significantly reduced and the number of mature DCs in the tumor were increased after A/D/I-dHMLB administration. Moreover, this nanomaterial induced not only the activation of both CD4+ and CD8+ T cells in the tumor site but also secretion of anti-cancer-related cytokines such as IL-12 and TNF-α [87]. Thus, co-delivery of anti-cancer and MDSCs-modulating agents using NPs can also be a promising therapeutic strategy.

## 5. Nanoparticles for Activating NK Cells

NK cells were identified in 1975 as innate lymphoid cells, which play an important role as modulator within the TME and directly kill cancer cells without any activating procedures such as complements or antibodies [141]. Particularly, NK-cell-mediated immune response is activated by the release of cytokines or chemokines, such as IFN-γ or TNFα, that can modulate an inflammatory response through various mechanisms. For example, Au et al. studied effective NK cells that are recruited and activated by epidermal growth factor receptor (EGFR)-targeted trivalent NPs [84]. EGFR-related NPs could target tumor cells that overexpress EGFR and also triggered NK cells-mediated anti-cancer immune responses. Exosomes, especially DC-derived exosomes (Dex), are a powerful activator of NK cells [86]. One study showed that Dex expressed both interleukin 15 receptor alpha subunit (IL-15Rα) and natural killer group 2-member D ligand, which promoted NK cell proliferation and directly induced NK cell activation [142]. In another approach, cationic nanoparticles (cNPs) were used to stimulate NK cells for the activation of anti-cancer immune responses in the triple-negative breast cancer model [81]. With treatment with cNPs, the cytotoxic activity of NK cells were shown to be increased and subsequently reduced tumor growth in the tumor mice model.

On the other hand, innate-like T lymphocytes, natural killer T (NKT) cells perceive glycolipid antigens presented by CD1d, MHC class I-like protein. Activation of NKT cells triggers rapid pro-inflammatory cytokine for immune modulation and leads to chemokine responses. NKT cells control downstream innate and adaptive immune responses against cancers by interacting with APCs. Many studies recently focused on the activation of NKT cells, owing to its properties as mediator between innate and adaptive immunities [143]. According to its characteristics, NKT cells are thought to be important immune cells in cancer immunotherapy to enhance anti-tumor effects. Therefore, studies using synthetic NPs for drug delivery systems and targeted therapeutic effects are needed.

## 6. Nanoparticles for Targeting Neutrophils

Neutrophils (also refer as neutrocytes), the most plentiful leukocytes, play a critical role in the regulation of invading infections and involved in innate immune responses. They respond to chemotaxis and can rapidly transmigrate into damaged or infected tissues such as TME.

Here we introduce several studies utilizing neutrophils for improving cancer therapeutic efficacy. One study developed albumin-based NPs that hitchhike activated neutrophils and promote immune responses in tumor sites [144]. Similarly, Li et al. reported a nano-pathogenoid system capable of in situ hitchhiking circulating neutrophils to enhanced migration towards tumors. In particular, cisplatin-loaded nanoplatform combined with photothermal therapy (PTT) exerted complete tumor removal in all treated mice [145]. The other study showed that the pre-treatment of cabozantinib (a multi-receptor tyrosine kinase inhibitor) increased the neutrophil-mediated carrying of PLGA-NPs coated with BSA into the prostate tumor [144]. Therefore, neutrophil-mediated drug delivery systems are a potential and promising strategy to promote NP accumulation and tumor infiltration in cancer therapy.

## 7. Challenges and Future Perspectives

Based on the knowledge and understanding of the immune cell dependent effects of traditional cancer therapy (chemo- and radiotherapy), cancer immunotherapy has rapidly emerged as a standard tool for comprehensive cancer care. Cancer immunotherapy is a promising anti-cancer treatment that attacks specific cancer cells by strengthening the immune system in our body, unlike well-established conventional cancer therapies targeting directly the malignant cells [146]. In particular, ICBs targeting checkpoint inhibitor molecules (e.g., CTLA-4, PD-1 and PD-L1) trigger the activation of exhausted cancer-specific T cells [147,148,149]. CTLA-4 is expressed by activated T cells and Treg cells, related with immune tolerance. T cells undergo anergic state upon binding to CD80 and CD86 expressed by APCs [150]. Similarly, PD-1 molecules on T cells that bind PD-L1/PD-L2 ligands expressed on cancer cells, lead to the inactivation of T-cell receptor signaling and T-cell exhaustion [148,149]. On the other hand, CAR-T cell therapy enables the transfer of plenty of anti-tumor T cells that were genetically modified to induce artificial T-cell receptors in tumor patients [151,152]. The durable clinical responses of these therapies were demonstrated in patients with several cancer types in recent years [153,154,155]; however, only ~30% of the patients, or a few tumor types with high mutational load, respond to these promising immunotherapies [156].

Note that the failure of current immunotherapies is closely related with the activity of innate immune cells within TMEs. Moreover, recent evidence underscores the importance of cross-talk between innate immune cells and cancer cells in controlling cancer progression, metastasis, and response to various forms of immunotherapy. Therefore, altering TMEs, specifically, targeting innate immune cells to selectively enhance cytotoxic T-cell activation and reduce MDSC/M2 migration, together with limiting the suppressive effects induced in TMEs, is likely to increase the current response rate of ICBs and other immunotherapies. Therefore, innate immunity is now highlighted as a potential combination-target for immune checkpoint therapy.

Here, we review the contribution of advanced nanotechnology for the modulation of innate immune cells in TMEs, their significance on current cancer therapies and the potentials for developing novel therapeutic strategies. Despite of recent pioneering research which has ushered in a new era of the nanomaterial-based modulation of innate immune cells in TME, several issues should be addressed before such applications can be translated into the clinical. First of all, given that drug-loaded NPs can evoke immune toxicity through interaction with immune cells, in-depth toxicity studies must be carefully examined. In addition, NPs that modulate innate immune cells should be designed to target only specific cells with fewer off-target effects and to provide a sustained anti-tumor effect to patients. Accumulating data on ligands that differentiate immune cells of tumors and normal tissues. For example, mannose/galactose receptors are present on both M1-type macrophages and M2-type TAMs. Therefore, to maximize the targeting ability of nanomaterials to TME-specific immune cells, additional research is required to discover more specific ligands for innate immune cells. Importantly, a precise understanding of the contribution of the innate immune systems to the anti-cancer immune responses will be a key feature to consider for the development of nanomaterial-based clinical strategies for cancer therapy. 

## Abbreviations

TME	Tumor Microenvironment
ECM	Extracellular Matrix
TAMs	Tumor-Associated Macrophages
DCs	Dendritic Cells
NKs	Natural Killer Cells
ADCC	Antibody-Dependent Cellular Cytotoxicity
ADCP	Antibody-Dependent Cellular Phagocytosis
CAR-T	Chimeric Antigen Receptor-T
MDSCs	Myeloid-Derived Suppressor Cells
IFN-γ	Interferon-γ
FOLR2	Folate Receptor-2
CaBP	Calcium Bisphosphonate
CSF-1	Colony-Stimulating Factor-1
MCSF	Macrophage Colony-Stimulating Factor
siRNA	Small Interfering RNA
CCL2	Chemokine Ligand 2
APCs	Antigen Presenting Cells
PBAE	Poly-Beta-Amino-Esters
IRF5	Interferon Regulatory Factor 5
BMDMs	Bone Marrow-Derived Macrophages
miRNAs	MicroRNAs
SIRPα	Signal-Regulatory Protein Alpha
MHC I	Major Histocompatibility Complex I
Th	Helper T Cell
Tc	Cytotoxic T Cell
TAA	Tumor-Associated Antigen
NPs	Nanoparticles
AuNPs	Gold Nanoparticles
LN	Lymph Nodes
CT	Computed Tomography
MSNs	Mesoporous Silica Nanoparticles
SA	Steric Acid
M-COSA	Mannose-engineered COSA
BMDCs	Bone Marrow-Drived Dendritic Cells
ELISA	Enzyme-Linked Immunosorbent Assay
CCR5	Chemokine Receptor type 5
Tregs	Regulatory T Cells
iNOS	Inducible Nitric Oxide Synthase
ROS	Reactive Oxygen Species
RNS	Reactive Nitrogen Species
ATRA	All-Trans Retinoic Acid
EGFR	Epidermal Growth Factor Receptor
Dex	Dendritic cells-derived exosomes
cNPs	Cationic Nanoparticles
NKT	Natural Killer T cells
PTT	Photothermal Therapy
